# Magnetostatic surface waves propagation at dissipative ferrite-MTMs-metal structure

**DOI:** 10.1186/2193-1801-2-584

**Published:** 2013-10-31

**Authors:** Zeyad I Al-Sahhar, Mohammed M Shabat, Hala J El-Khozondar

**Affiliations:** Physics Department, Al-Aqsa University, Gaza, Palestine; Physics Department, Islamic University, Gaza, Palestine; Electrical Engineering Department, Islamic University, PO Box 108, Gaza, Palestine

**Keywords:** Metamaterials, Ferrite, Magnetostatic surface waves, Surface waves

## Abstract

The magnetostatic surface waves (MSSW) propagation in a layered structure composed of ferrite film covered by air and on top of metamaterial (MTM) placed on metal is discussed. Dispersion equations which relate the parameters of different layers are derived and used to analyse propagation of MSSW. It is found that the MSSW excitation band depends on the thickness of the MTM layer and ferrite layer.

## Introduction

Metamaterial (MTM) is a manmade material with negative value for both permittivity and permeability at a certain frequency. For wave propagating in MTMs, the electric field, the magnetic field, and wave vector follow the left-hand rule, rather than the usual right-hand rule. MTMs unusual electromagnetic phenomena such as a sign variation of group velocity, negative refraction, and perfect lensing have been theoretically studied by Veselago ( 1968). Pendry et al. achieved experimentally negative permittivity material by using metallic wires ( 1996) and negative permeability material from a periodic arrangement of split ring resonators (SRRs) ( 1999) in the GHz band. MTMs photonics are associated with new concepts and potential applications (Pendry 2000, Smith et al. 2004).

During the last decade, slab MTM structures gained significant interest (Li and Ma 1999, Reuben 1999, Moses and Engheta 2001, Rybin and Raza 2009). A substantial amount of research has been conducted to study MTMs applications in communication such as isolators (El-Khozondar et al. 2008a) and sensors (El-Khozondar et al. 2008b).

Ferrites are magnetic materials characterized by anisotropic properties and various energetic interactions, such as dipole, exchange magnetoelastic and magnetooptical. In the microwave range, Ferrites permeability operates in magnetically saturated states that vary with the saturation magnetization, the microwave frequency, and the outside magnetic field (Snoek 1947). The propagation of magnetostatic waves in layered structures consisting of ferrite materials (Kee et al. 2000, Damon and Eshbach 1961, Bongianni 1972, Bestler 1959, Courtois et al. 1970) and ferrite-MTMs (El-Khozondar et al. 2010, Mansour et al. 2009, Al-Sahhar et al. 2013) have attracted much attention owing to various applications of ferrite in the microwave devices and are extremely important for designing integrated devices such as narrow-frequency optical or microwave filters and high-speed switches (Vasseur et al. 1996, Al-Wahsh et al. 1999, Gulyaev and Nikitov 2001).

In this paper, the transverse electric (TE) wave propagation in a structure consists of MTM film surrounded by a metal substrate and a magnetized ferrite cover bounded by air is investigated. In the following section, the theoretical steps are summarized. The numerical analysis and results are discussed in section Numerical analysis and results. The last section is dedicated for the conclusion.

## Theoretical analysis

**Figure 1:**
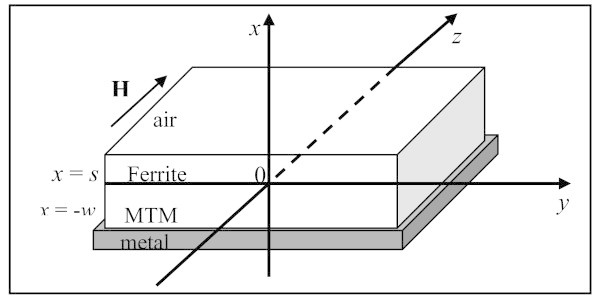
**Asymmetric waveguide structure consists of a Ferrite film having width** ***s*** **surrounded from above by air and from below by a dissipative MTMs layer with width** ***w*** **placed on a metal substrate.**

Figure 1 exhibits the configuration of an asymmetric planar waveguide that consists of dissipative MTMs film having width *w* placed on a metal substrate and bounded from above by a Ferrite film with width *s* covered by air.

The dissipative MTM film has both permittivity *ε*_*M*_ and permeability *μ*_*M*_ depending on the frequency (*ω*) as follows:
12

where *ω*_*r*_ is the resonance frequency, *ω*_*p*_ is the plasma frequency and *γ* is the damping factor. The values of the parameters *ω*_*r*_, *ω*_*p*_ and *F* are chosen to fit the experimental data (Shebly 2001) : *ω*_*p*_ = 26.6π GHz, and *F* = 0.37. In the ferrite slab, a static magnetic field is applied in the *z* direction, resulting in a uniform intensity *H*_0_ within the ferrite. The ferrite slab has positive permittivity *ε*_*f*_ and permeability *μ*_*f*_ defined as (Bestler 1959, Courtois et al. 1970, El-Khozondar et al. 2010, Mansour et al. 2009, Al-Sahhar et al. 2013)
3

where , , *μ*_*1*_ = 1, *ω* is surface wave frequency, *ω*_*H*_ = *σH*_0_ is the Larmor frequency, *ω*_*M*_ = *4π σ M*_*0*_ is the magnetic frequency, *σ* is the electromagnetic oscillation frequency, *4πM*_*0*_ is the ferrite saturation magnetization, and *H*_0_ is the applied magnetic field (Bespyatykh et al. 2001).

We assumed transverse electric fields (TE) propagating in the y direction such that ***E*** = (0, 0, *E*_*z*_) e^jωt^ and ***H*** = (*H*_*x*_, *H*_*y*_, 0) e^jωt^. The field equations are obtained by applying the TE fields into Maxwell’s equations as follows:
4

Where the variation with *z*-direction is assumed to be zero, *i* indicates *f* for Ferrite layer, *M* for MTMs layer, and *l* for the linear cladding layer (air), *q*_*l*_ = *k*_0_, , *q*_*M*_ = *k*_0_*ε*_*M*_, *k*_0_ = *ω*/*c* and , which is called the Voigt permeability. The dispersion equation (5) is derived by applying boundary conditions to the solutions of equation (4).
5

where  and *k*_*xi*_ and *k*_*y*_ are the components of the wave vector directed along the coordinate. The dispersion relation equation (5) relates the transverse wave numbers for each media. It is an implicit equation that gives the surface wave dispersion relation.

In the calculations, two limits are considered: *w* → ∞ which simplifies the structure to air-ferrite -MTM and *w* = 0 which reduces the structure to air-ferrite-metal.

At the limit *w* → ∞, equation (5) becomes,
6

While at the limit *w* = 0, equation (5) reduces to the following form,
7

Equation (5) to equation (7) can only be solved numerically. The solutions of these equations give the MSSW at the different limits. The limiting frequencies for MSSW free ferrite film are expressed as follows (Damon and Eshbach 1961):
89

where *ω*_*s*_ is the starting frequency and *ω*_*fin*_ is the final frequency.

## Numerical analysis and results

**Figure 2:**
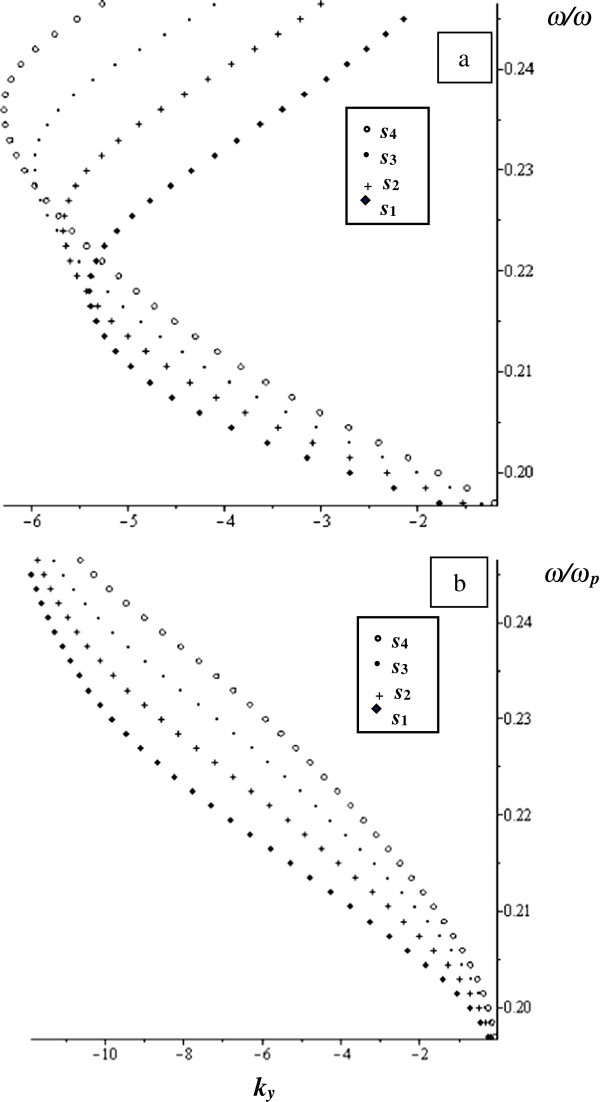
**The normalized frequency versus** ***k***_***y***_ **at** ***w*** **= 790 μm and** ***γ*** **= 0.1 for different values of** ***s*** **as indicated in the figure. a)** real part and **b)** imaginary part.

The dispersion equations (5, 6 and 7) are solved numerically to get information about MSSW at different limits. We chose *ω*_*H*_ = 1.76 × 10^7^H_0_ rad/s, *ω*_*M*_ = 1.76 × 1870H_0_ rad/s, *H*_0_ = 367 Oe, *ε*_*2*_ = 15, and *ω*_*p*_ = 83.56 × 10^9^ rad/s, *ω*_*s*_/*ω*_*p*_ = 0.19 and *ω*_*fin*_/*ω*_*p*_ = 0.27. The value of frequency *ω* is chosen such that *ε*_*M*_, *μ*_*M*_, and *μ*_*v*_ are negative. The normalized MSSW frequency with respect to plasma frequency (*ω*/*ω*_*p*_) is plotted as a function of the y component of the propagation constant (*k*_*y*_) as shown in Figure 2.

The parameter values are: *w* = 790 μm, *γ* = 0.1, and the ferrite thickness *s* varies as follows: *s*_*1*_ = 6.2 μm, *s*_*2*_ = 7.2 μm, *s*_*3*_ = 8.2 μm and *s*_*4*_ = 9.2 μm. It is noticed from Figure 2(a) that the real part of the normalized frequency curve has similar behaviour to the unbounded ferrite film in which MSSW appears at almost the same range of frequencies, 0.1965 to 0.2333. However, in Figure 2(a), there is no curve in the + *k*_*y*_ that exists in the case of unbounded ferrite film and which would have been symmetric to the existing curve regarding to the frequency axis. That is, in the current structure, the MSSW is always directed oppositely to + *k*_*y*_-axis, i.e. this MSSW is unidirectional and backward. Moreover, in Figure 2(a), MSSW exhibits a bierfringent behaviour where at certain values of *k*_*y*_, two values of frequencies are allowed to pass. The imaginary parts of the normalized frequency which comes due to the complex behaviour of the MTMs parameters are displayed in Figure 2(b). The wave also propagates in one direction which is opposite to + *k*_*y*_-axis without bierfringent behaviour. The bierfringent behaviour does not appear in the unbounded ferrite film characteristic curve or in the similar structure free of loss (Al-Sahhar et al. 2013). The MSSW behaviour depends on *s*.

**Figure 3:**
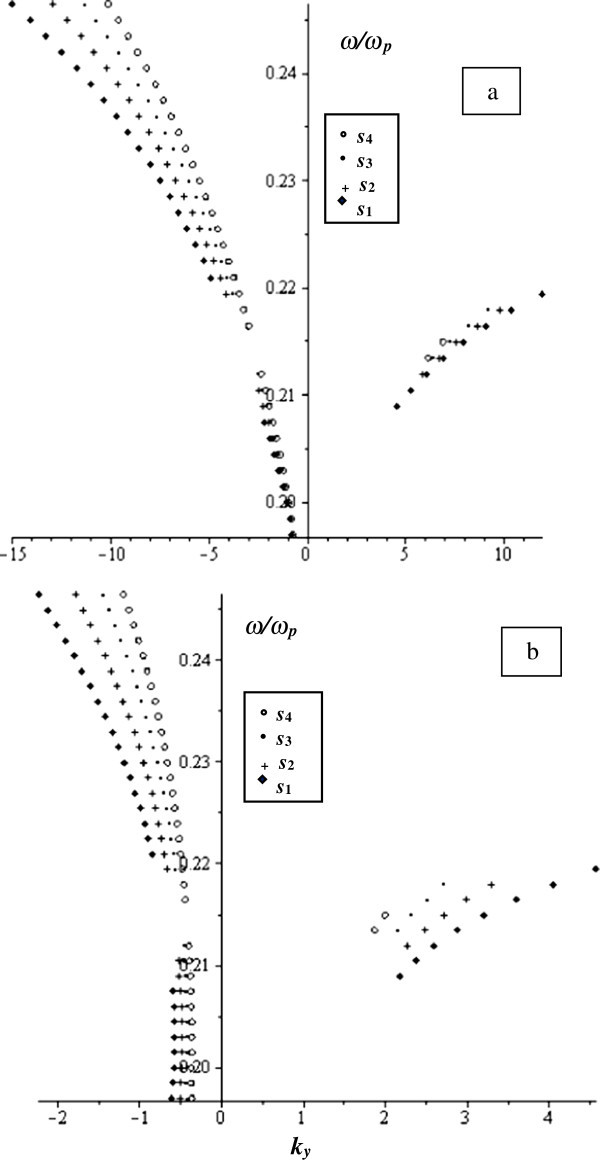
**The normalized frequency versus** ***k***_***y***_ **at** ***w*** **= 790 μm and** ***γ*** **= 0.05 for different values of** ***s*** **as indicated in the figure. a)** real part and **b)** imaginary part.

Decreasing the value of the damping factor to the value, *γ* = 0.05, and keeping all the other parameters constant, we get the normalized frequency as a function of *k*_*y*_ as displayed in Figure 3. In both the real part (Figure 3a) and the imaginary part (Figure 3b), we observe that the MSSW propagates opposite to the + *k*_*y*_-direction indicating that the MSSW is unidirectional and the bierfringent behaviour disappears. Moreover, the values of the normalized frequency of MSSW vary as *s* changes.

**Figure 4:**
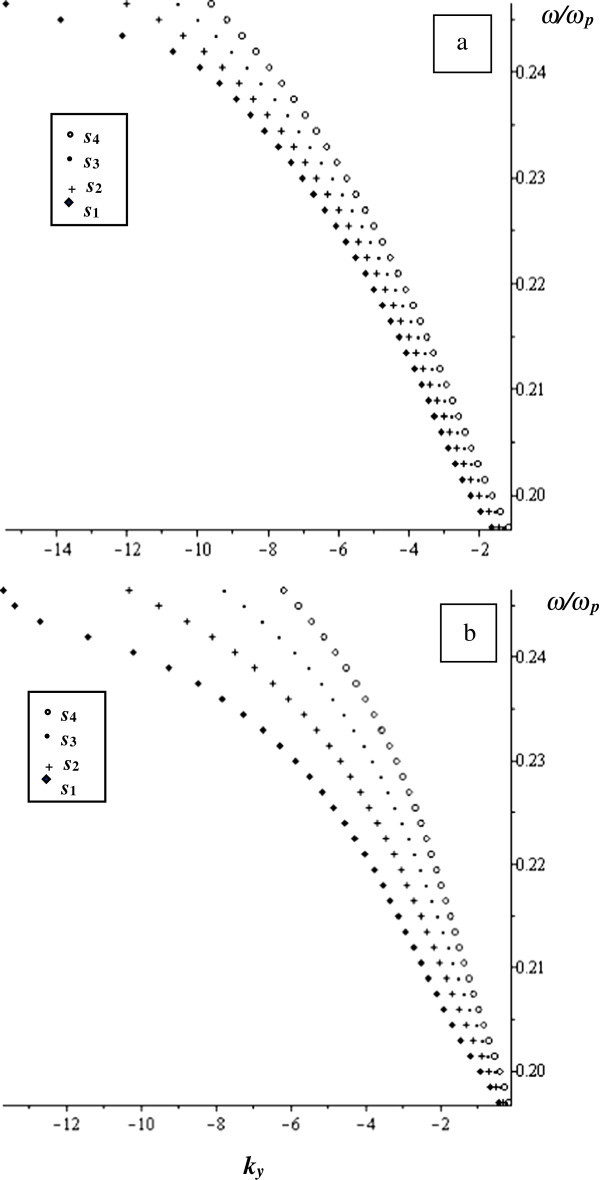
**The normalized frequency versus** ***k***_***y***_ **at** ***w*** **= 790 μm and** ***γ*** **= 0.01 for different values of** ***s*** **as indicated in the figure. a)** real part and **b)** imaginary part.

Taking the smaller value of *γ* = 0.01 and keeping all the other parameters unchanged, we get the normalized frequency as a function of *k*_*y*_ as illustrated in Figure 4. In both the real part (Figure 4a) and the imaginary part (Figure 4b), we see that the MSSW propagates in both directions (±y) in agreement with the characteristic curve for unbounded ferrite film. We also notice that the wave either propagates in one direction or in the other at certain ranges of frequencies. The MSSW behaviour changes as *s* changes.

From Figures 2, 3, 4, we see that as *γ* decreases, the behaviour of the MSSW approaches the behaviour of the structure with omission of loss (Al-Sahhar et al. 2013). We also see that as the ferrite layer thickness, s, changes, the MSSW undergoes different behaviour and the corresponding frequency to a certain propagation constant *k*_*y*_ changes.

**Figure 5:**
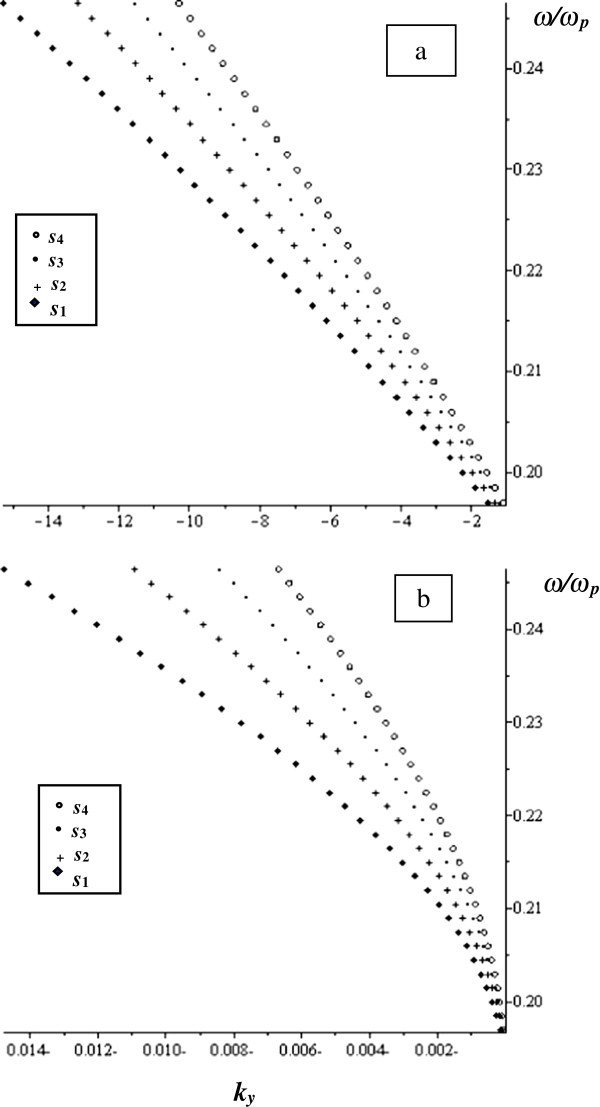
**The normalized frequency versus** ***k***_***y***_ **at** ***w*** **= 0.790 μm and** ***γ*** **= 0.1 for different values of** ***s*** **as indicated in the figure. a)** real part and **b)** imaginary part.

To understand the effect of the thickness of the MTM layer on the behaviour of the MSSW, we plot in Figure 5 the normalized frequency versus *k*_*y*_ at *w* = 0.790 μm, *γ* = 0.1 and all the other parameters are kept the same. Figure 5 shows that as the thickness of MTM changes, MSSW normalized frequency loses the bierfringent behaviour. However, it stays unidirectional. That is the waves propagates in–*k*_*y*_. It also changes as *s* varies.

**Figure 6:**
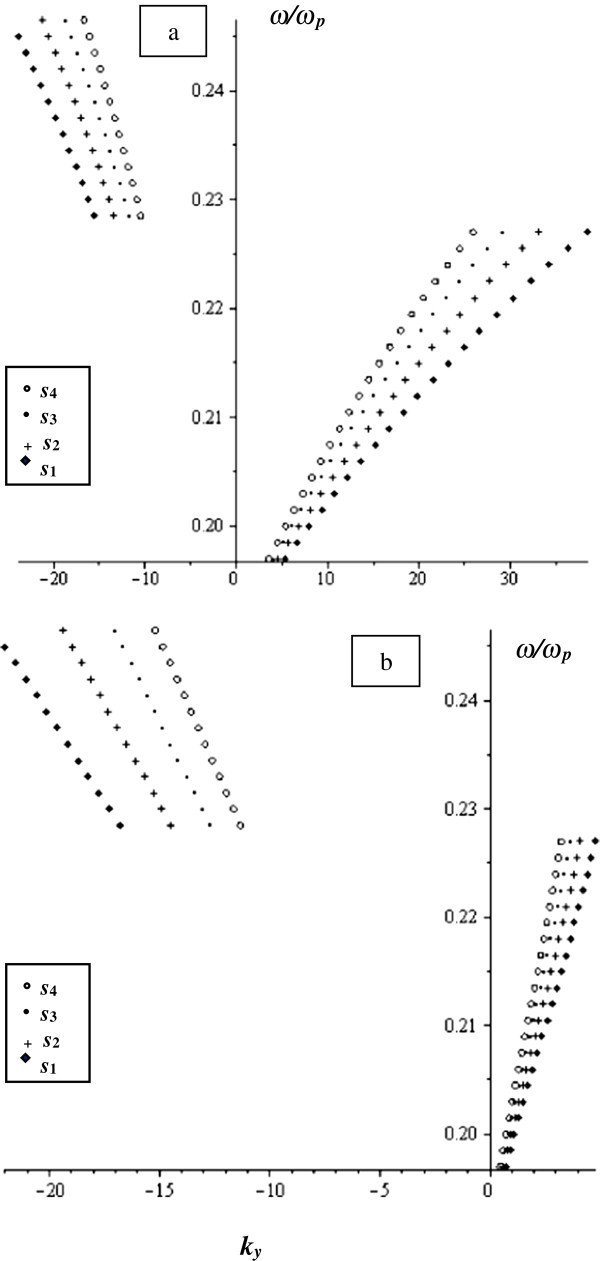
**The normalized frequency as a function of** ***k***_***y***_ **in the limit** ***w*** **→ ∞ at** ***γ*** **= 0.1 for different values of** ***s*** **as indicated in the figure. a)** real part and **b)** imaginary part.

Figure 6 exhibits the relation between *ω*/*ω*_*p*_ and *k*_*y*_ at the limit *w* → ∞ at *γ* = 0.1, and *s* varies as follows: *s*_*1*_ = 6.2 μm, *s*_*2*_ = 7.2 μm, *s*_*3*_ = 8.2 μm and *s*_*4*_ = 9.2 μm. It is shown that MSSW propagates in both directions ± *y*. However, it propagates in one or the other direction at certain ranges of frequencies. It also changes as *s* changes.

**Figure 7:**
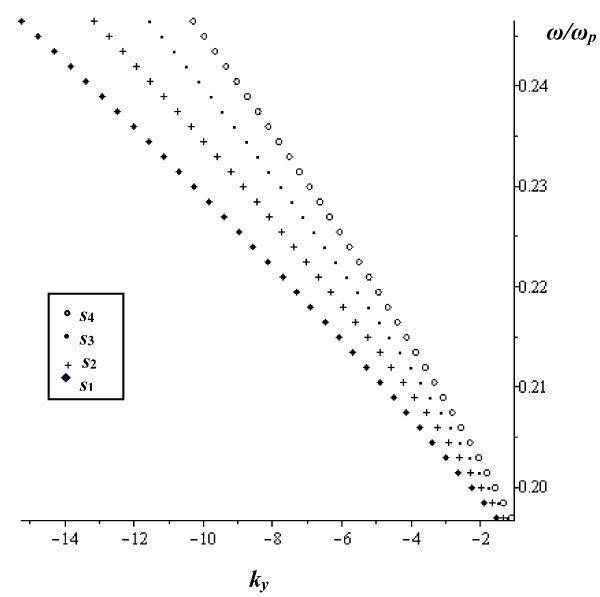
**The normalized frequency as a function of** ***k***_***y***_ **at the limit** ***w*** **= 0 at** ***γ*** **= 0.1 for different values of** ***s*** **.**

The normalized magnetostatic waves frequency, *ω*/*ω*_*p*_, is plotted as a function of the propagation constant at the limit *w* = 0 at *γ* = 0.1, and *s* varies as follows: *s*_*1*_ = 6.2 μm, *s*_*2*_ = 7.2 μm, *s*_*3*_ = 8.2 μm and *s*_*4*_ = 9.2 μm in Figure 7. In this case, only backward MSSW propagates and its normalized frequency changes as *s* varies.

## Conclusion

MSSW propagation is studied in an asymmetric slab waveguide consisting of ferrite film sandwiched between air and MTM film placed on a metal substrate. The dispersion relation for the MSSW is studied at three different cases corresponding to the different MTM layer thicknesses: finite thickness, infinite thickness, and zero. The three resulted dispersion equations are numerically solved. Results are presented by plotting the MSSW frequency as a function of the y- component of the propagation constant *k*_*y*_. Results demonstrate that MSSW frequency depends on both MTM layer and ferrite layer thicknesses. It is found that MSSW might travel in unidirectional–*k*_*y*_ or in both direction ± *y* depending on the damping factor, MTMs thickness, and/or ferrite thickness. This result is promising in improving the waveguide performance. It also has industrial applications; *i. e*. isolators and sensors.

## Authors’ information

Zezad I. Al-Sahhar was born in 1961 in Jabalia, Gaza, Palestinian Territory. He got his B.Sc. in Physics in 1984 from King Abdulaziz University at Kingdom of Saudi Arabia. He earned his M.Sc. in Physics in 2001 from Islamic University of Gaza, Gaza, Palestine and his Ph.D. in Physics in 2006 from Ain Shams University, Cairo, Egypt. He has awarded research assistant at Saudi Arabian National Center for Science and Technology at the Kingdom of Saudi Arabia during the period from 1984 to 1985. In the period 1988–1991, he worked as teacher at high schools in Gaza. In 1991, he worked as a lecturer in Physics Department at College of Education, Gaza, Palestinian Territory. In 1999, he became a member of Physics Department at Al-Aqsa University, Gaza, Palestinian Territory. Then, he was promoted to assistant professor in 2006. He became a head of Physics Department in the period from 2008 to 2009. He participated in several conferences and workshops. His research interests are: non-linear optical sensors, magneto static surface waves, numerical methods, photovoltaic cells and metamaterial application.

Mohammed M. Shabat was born in Beit Hanoun, Gaza Strip, Palestinian Territory in 1960. He received his B.Sc. in Physics from Al-Aazhar University, Cairo, Egypt in 1984 and the Ph.D. degree from the University of Salford, UK in 1990. He was a research fellow at the University of Manchester Institute of Science and Technology, UK, from 1989 to 1992. In April 1992, he joined the Physics Department at the Islamic University of Gaza (IUG) as an assistant professor of Physics. He became an associate professor in 1996 and a professor of Physics in 2000. In the period 2001–2005, he was the vice president for Administrative Affairs at IUG. He was awarded the Shoman Prize for a Young Arab Scientist (Jordan) in 1995, and the Humboldt Research Fellowships in 1998–1999 at the Center of Semiconductor Technology and Optoelectronics, Duisburg-Essen University, Germany. He was a visiting scientist at Bochum University, Germany in 1994; at the Institute National Polytechnic de Grenoble (INPG), France in 1995; at Salford University, UK in 1997; ICTP, Trieste, Italy in 1996, 1997 and 1998, 2000, 2001, 2003, 2004; and Duisburg-Esse University, Germany in 1998, 1999 and 2002, 2003, 2004 and 2006. Professor Shabat has received Galileo Galilei Award of the International Commission of Optics (affiliated to ICSU and IUPAP) in 2006. His research interests include nonlinear optical sensors, optoelectronics, magnetostatic surface waves, numerical techniques, mesoscopic systems, energy, applied mathematics, nanotechnology, and physics education.

Hala J. El-Khozondar was born in Gaza, Palestinian Territory. She got her B.Sc. in Physics from BirZeit University, Palestinian Territory in 1987. She earned her Ph.D. in physics from New Mexico State University (NMSU), USA in 1999. She joined the physics faculty at BirZeit University in 1987. She had a Postdoc award at Max Planck Institute in Heidelberg, Germany in 1999. In 2000, she worked as assistant professor in the electrical engineering (EE) department at Islamic University of Gaza. In 2007, she was promoted to associate professor EE department. She is now a full professor at EE department and a fellow for the world academy of science (FTWAS) and for the Arab region FTWAS-ARO. She worked in initiating and developing the quality assurance unit and the external relations at Islamic University of Gaza. She advises several graduate and undergraduate students. She participated in several conferences and workshops. Her research interests are focused on studying Wireless communication, optical communication, nonlinear optics, optical fiber sensors, magneto-optical isolators, optical filter, MTMs devices, biophysics, electro-optical waveguides, and numerical simulation of microstructural evolution of polycrystalline materials. She has several publications in highly ranked journals including Journal of Light Wave Technology, IEEE Journal of Quantum Electronics and Optics Letter. She is a recipient of international awards and recognitions, including a Fulbright Scholarship, DAAD short study visit, a Alexander von Humboldt-Stiftung Scholarship, Erasmus Mundus, and the Islamic University Deanery Prize for applied sciences. She is also coordinator for several projects including TEMPUS for promoting long life education, and Al-maqdisi to enhance collaboration with French partners.
